# The interplay between *XPG*‐Asp1104His polymorphism and reproductive risk factors elevates risk of breast cancer in Tanzanian women: A multiple interaction analysis

**DOI:** 10.1002/cam4.4914

**Published:** 2022-06-12

**Authors:** Ismael C. Adolf, Linus P. Rweyemamu, Gokce Akan, Ted F. Mselle, Nazima Dharsee, Lucy A. Namkinga, Sylvester L. Lyantagaye, Fatmahan Atalar

**Affiliations:** ^1^ University of Dar es Salaam Mbeya College of Health and Allied Sciences Mbeya Tanzania; ^2^ University of Dar es Salaam Department of Molecular Biology and Biotechnology Dar es Salaam Tanzania; ^3^ Muhimbili University of Health and Allied Sciences MUHAS Genetic Laboratory, Department of Biochemistry Dar es Salaam Tanzania; ^4^ Near East University DESAM Research Institute Nicosia Cyprus; ^5^ Ocean Road Cancer Institute Academic, Research and Consultancy Unit Dar es Salaam Tanzania; ^6^ Istanbul University Child Health Institute, Department of Rare Diseases Istanbul Turkey

**Keywords:** breast cancer, DNA repair genes, multifactor dimensionality reduction, polymorphism, *XPG*

## Abstract

**Background:**

Reproductive history and genetics are well‐known risk factors of breast cancer (BC). Little is known about how these factors interact to effect BC. This study investigated the association of ten polymorphisms in DNA repair genes with BC susceptibility in the Tanzanian samples and further analyzed the association between reproductive risk factors and disease risk

**Methods:**

A hospital‐based case–control study in 263 histopathological confirmed BC patients and 250 age‐matched cancer‐free controls was carried out. Allelic, genotypic, and haplotype association analyses were executed. Also, multifactor dimensionality reduction (MDR), and interaction dendrogram approaches were performed.

**Results:**

The frequency of genotypic and allelic variants of *XRCC1*‐Arg399Gln (rs25487), *XRCC2*‐Arg188His (rs3218536), *XRCC3*‐Thr241Met (rs861539), *XPG*‐Asp1104His (rs17655), and *MSH2*‐Gly322Asp (rs4987188) were significantly different between the groups (*p* < 0.05). Moreover, *XRCC1*‐Arg399Gln (rs25487), *XRCC3*‐Thr241Met (rs861539), and *XPG*‐Asp1104His (rs17655) were associated with the increased risk of BC in co‐dominant, dominant, recessive, and additive genetic‐inheritance models (*p* < 0.05). *XRCC1*‐Arg/Gln genotype indicated a 3.1‐fold increased risk of BC in pre‐menopausal patients (*p* = 0.001) while *XPG*‐His/His genotype showed a 1.2‐fold increased risk in younger BC patients (<40 years) (*p* = 0.028). Asp/His+His/His genotypes indicated a 1.3‐fold increased risk of BC in PR+ patients and a 1.1‐fold decreased risk of BC in luminal‐A patients (*p* = 0.014, *p* = 0.020, respectively). MDR analysis revealed a positive interaction between BC and the *XPG*‐Asp1104His (rs17655) together with family history of cancer in the first‐degree relatives. Dendrogram analysis indicated that the *XPG*‐Asp1104His (rs17655) and family history of cancer in first‐degree relatives were significantly synergistic and might be associated with an elevated risk of BC in Tanzania.

**Conclusions:**

The *XPG*‐Asp1104His (rs17655) might exert both independent and interactive effects on BC development in the Tanzanian women.

## Graphical abstract

This study investigated the association of ten polymorphisms in DNA repair genes with breast cancer susceptibility in the Tanzanian women, and further analyzed the association between reproductive risk factors and disease risk. Our findings indicated that the XRCC1 rs25487, XPG rs17655, XRCC3 rs861539, and hMSH2 rs4987188 were associated with breast cancer risk in the Tanzanian women. To the best of our knowledge, this is the first study to demonstrate the XPG rs17655 polymorphism interaction with reproductive factors, and with histopathological characteristics of breast tumors and family history of cancer in the first‐degree relatives.

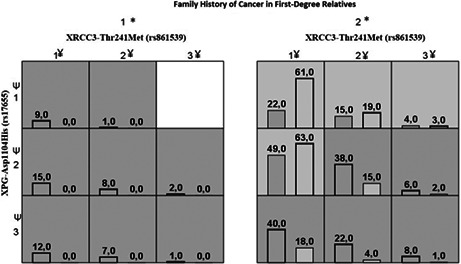

## 1 INTRODUCTION

Breast cancer (BC) is the most common malignancy among women worldwide.^1^ Various factors including genetic, reproductive, environmental, and lifestyle are well described BC risk factors that contribute enormously to the disease.^2^ Although the mechanism underlying the disease has not been fully elucidated, DNA damage by exogenous and endogenous agents are reported to result in failure of maintaing the genome integrity, hence, induce the BC.^3^, ^4^, ^5^ Moreover, various reproductive risk factors such as the age at first full‐term pregnancy, nulliparity, breast‐feeding, and family history of cancers are also reported to increase the risk of BC individually.^6^

Cells are endowed with the DNA repair systems that identify and correct the damaged DNA portion, thereby preventing the damage's carcinogenic effect.^7^, ^8^ There are four main DNA repair pathways: the base excision repair (BER), the nucleotide excision repair (NER), the mismatch repair (MMR), and the double strand break repair (DSBR).^8^ The choice of the repair pathway to engage is subject to the nature of the damaging agent, and the extent of the DNA damage.^3^, ^8^

It is apparent that the polymorphisms of the DNA repair genes can compromise the DNA repair capacity, allowing the accumulation of carcinogenic mutations. Many polymorphisms residing in the DNA repair genes were investigated in various populations to understand their association with the risk of developing different cancers including BC.^9^, ^10^ However, there is no consensus result yet for most of these polymorphisms, and this could be partially explained by ethnic and geographic dynamics. Amid these conflicting findings, there is a need to investigate the polymorphisms as putative genetic markers in the understudied populations of developing countries like Tanzania, where BC is mostly diagnosed at late stages.^11^, ^12^ Understanding the genetic markers predisposing people to develop BC and its association with reproductive risk factors are of paramount significance in identifying people at high risk. This would enable early diagnosis and treatment of BC, ultimately leading to reduced mortalities.

Therefore, this study aimed to examine the association of BC with the DNA repair genes polymorphisms of the X‐ray repair cross‐complementing 1 (*XRCC1*‐Arg399Gln; rs25487), the apurinic/apyrimidinic endonuclease 1 (*APE1*‐Asp148Glu; rs1130409), the human 8‐oxoguanine DNA glycosylase (*hOGG1*‐Ser326Cys; rs1052133), the xeroderma pigmentosum group G (*XPG*‐Asp1104His; rs17655), the xeroderma pigmentosum group D (*XPD*‐Lys751Gln; rs13181), the X‐ray repair cross‐complementing 2 (*XRCC2*‐Arg188His; rs3218536), the X‐ray repair cross‐complementing 3 (*XRCC3*‐Thr241Met; rs861539), the *RAD51*‐4719A/T; rs2619679, the *RAD51*‐4601A/G; rs5030789, and the human MutS homolog 2 (*hMSH2*‐Gly322Asp; rs4987188). Also, the association of selected polymorphisms with reproductive factors and their contribution to BC development in Tanzanian women was investigated.

## 2 METHODS

### 2.1 Study population

A total of 263 women BC patients treated at the Ocean Road Cancer Institute (ORCI) in Dar es Salaam between 2019 and 2021 constituted the patients group. The eligibility criteria for a patient were: must be having a complete immunohistochemistry (IHC) data (ER, PR, and HER‐2 statuses), and the disease confirmed by histological examination and verified by a pathologist. The BC samples were histologically and pathologically examined at Muhimbili National Hospital (MNH), Bugando Medical Center (BMC), or Kilimajaro Christian Medical Center (KCMC). Tumor biomarkers including ER, PR, and HER2/neu were determined by immunohistochemistry upon the formalin fixed paraffin‐embedded blocks of BC tissues as described elsewhere.^13^

The control group was composed of 250 age‐group matched cancer‐free women who voluntarily attended the ORCI facility for cancer screening programs. Subjects with previous history of cancer and psychiatric diseases were exluded from the study. All BC patients and controls were of Tanzanian origin. Demographic, clinical characteristics (for patients) and reproductive factors such as menopausal status, parity, breast‐feeding, etc. were recorded from both groups. The study was approved by the Institutional Review Board of the ORCI, and the Ethics Committee of the Tanzania National Institute for Medical Research (NIMR). Each participant gave a written consent.

### 2.2 DNA extraction and genotyping

Peripheral blood samples were collected from all the participants. Genomic DNA was isolated from blood leucocytes using a High Pure PCR Template Preparation Kit (Roche, Diagnostics GmbH, Mannheim, Germany), as per manufacturer's recommendations. Genotyping of *XRCC1* rs25487, *XPD* rs13181, *APE1* rs1130409, *XRCC2* rs3218536, *XRCC3* rs861539, *hOGG1* rs1052133, *XPG* rs17655, *hMSH2* rs4987188, *RAD51* rs2619679 and *RAD51* rs5030789 polymorphisms (Table 1) was performed with the LightSNiP typing assay with SimpleProbe® (TIBMolBiol) using the Quantitative Real‐Time Polymerase Chain Reaction (QRT‐PCR). Genotyping of ten SNPs was carried out according to the melting curve analysis. The genotypes of samples were detected with different temperature profiles in one of the two peaks obtained.

**TABLE 1:** Studied single nucleotide polymorphisms (SNPs)

Repair pathway genes	Gene location	SNP (rs no.)	Base change	Mutant allele frequency AFR
Base excision repair				
*XRCC1*	19q13	rs25487	A/G	0.110
*APE1*	14q11	rs1130409	G/T	0.679
*hOGG1*	3p25	rs1052133	C/G	0.155
Nucleotide excision repair				
*XPG*	13q33	rs17655	G/C	0.501
*XPD*	19q13	rs13181	G/T	0.808
Repair of DNA double‐strand breaks				
*XRCC2*	7q36	rs3218536	A/G	0.008
*XRCC3*	14q32	rs861539	C/T	0.191
*RAD51*	15q15	rs2619679 rs5030789	A/T G/A	0.341 0.297
DNA mismatch repair				
*hMSH2*	2p21	rs4987188	G/A	0.002

*Note*: Mutant allele frequencies collected from 1000 Genomes project phase 3 (https://www.internationalgenome.org).

Abbreviation: AFR, African population.

### 2.3 Statistical analyses

All statistical analyses were performed using the SPSS software (Statistical Package for the Social Sciences, SPSS Inc, version 25). Genotype and allelic frequency distribution of polymorphisms between BC patients and controls were compared using Chi square (*χ2*) and Hardy–Weinberg equilibrium (HWE) and assessed by Fischer's exact test. Normally and abnormally distributed continuous variables were compared using the Student's *t*‐test and the Mann–Whitney *U*‐test, respectively, and the variables are expressed as mean ± Standard Deviation (SD). Categorical variables were compared using the (*χ* ^*2*^) test and results were given as percentages. Haplotypes were generated from the genotyped data and haplotype analysis was performed using Haploview 4.2. Odds ratios (ORs) and 95% confidence intervals (95% CI) for various genotypes were estimated by logistic regression analysis after adjustment of family history of cancer in the first‐degree relatives and breast‐feeding, as these two characteristics were significant among patients and controls. OR and 95% CI were estimated by binary logistic regression analysis adopting codominant, dominant, recessive and additive inheritance models. Akaike's information criterion (ACI) was used to choose the inheritance model that best fits the data. The significance level was defined when *p* < 0.05.

To assess the potential interactions of the DNA repair genes polymorphisms (gene–gene), and other disease‐associated factors (gene‐family history of cancer in the first‐degree relatives), multifactor dimensionality reduction (MDR version 3.0.2), which is a promising data‐mining with open‐source approach, was used. The MDR analysis aims to identify the overall best combination of all diseases associated factors that were found in the study, and evaluates the accuracy of each best model in the context of ten‐fold cross‐validation by the use of the Bayes classifier. The best model was extracted after the cross‐validation consistency measures the number of times in ten divisions of the dataset and it has maximal testing accuracy and cross‐validation consistency simultaneously. The corrections were done using the permutation testing by repeating the entire analysis on a thousand datasets that are consistent with the null hypothesis by MDR software. The interaction dendrogram and graph were also created by MDR 3.0.2 software.

## 3 RESULTS

### 3.1 Characteristics of the study group

A total of 263 BC patients and 250 controls were included in the study. The mean age of the BC patients was 49.3 ± 12.9) ranging from 26 to 81 years, while that of the control group was 49.9 ± 11.12 ranging from 26 to 80 years. Invasive ductal breast cancer of no special type (IDC‐NST) accounted for 89% of the BC patients, followed by 4.2% invasive lobular carcinoma (ILC), 1.9% mucinous carcinoma (MC) and 4.9% other type of histological tumor type. About 55.5% of patients had stage III, 27.4% stage IV, and 15.6% stage II breast carcinoma.

Immunohistochemical data revealed that among 263 BC patients, 64.3% expressed estrogen receptor (ER+), 52.9% progesterone receptor (PR+) and 35.7% overexpressing HER2 (HER2+) (Table 2). In terms of molecular subtypes, BC patients were classified as Luminal‐A (44.5%), Luminal‐B (22.4%), Triple‐negative breast cancer (TNBC) (22.1%), and HER2 enriched (11%) (Table 2).

**TABLE 2:** Immunohistochemical characteristics of the BC patients

Characteristics	BC patients (%) (*n* = 263)
ER	
+	64.3
−	35.7
PR	
+	52.9
−	47.1
HER‐2	
+	35.7
−	64.3
Molecular subtype	
Luminal‐A	44.5
Luminal‐B	22.4
HER‐2 enriched	11
TNBC	22.1

Abbreviations: BC, Breast cancer; ER, Estrogen receptor; HER‐2, Human epidermal growth factor receptor‐2; PR, Progesterone receptor; TNBC, Triple‐negative breast cancer.

Table 3 presents the distribution of selected demographic characteristics and reproductive risk factors of the BC patients and controls. The two groups were well‐matched for age (49.3 ± 12.9 years and 49.9 ± 11.2 years for BC patients and controls, respectively). There was no difference between the groups regarding BMI (27.8 kg/m^2^ and 28.1 kg/m^2^ for BC patients and controls respectively), mean age at menarche, at menopause, at first birth, smoking and alcohol consumption. BC patients having a family history of cancer in the first‐degree relatives and the breastfeeding ratio were significantly different between BC patients and controls (Table 3).

**TABLE 3:** Demographic and clinical characteristics of the BC patients and controls

Characteristics	BC patients (*n* = 263)	Controls (*n* = 250)	*p*‐Value
Age, years^b^	49.3 ± 12.9	49.9 ± 11.2	0.576
BMI^b^	27.8 ± 6.9	28.1 ± 5.7	0.942
Age at menarche, years^b^	14.8 ± 1.5	14.6 ± 1.2	0.157
Age of first birth, years^b^	22.8 ± 5.3	22.4 ± 4.6	0.558
Breastfeeding^a^			
Yes	76	92	**0.001**
No	24	8	
Age at menopause, years^b^	46.2 ± 7.5	47 ± 5.7	0.576
Pre‐menopause^a^	45	48	
Post‐menopause^a^	55	52	0.553
Family history of cancer in first‐degree relatives^a^			
Yes	21	0	**0.001**
No	79	100	
HRT^a^			
Yes	2	0	0.696
No	98	100	
Smoking^a^			
Yes	2	1	0.542
No	98	99	
Alcohol consumption^a^			
Yes	18	16	0.348
No	82	84	

*Note*: The *p*‐value ≤0.05 considered as statistically significant (in bold).

Abbreviations: BC, Breast Cancer; BMI, Body Mass Index; HRT, Hormone replacement therapy.

^a^ The values are calculated using the chi‐square test and the data are given in percentages.

^b^ The values are calculated using Student *t*‐test nand the data are given mean ± standard deviation.

### 3.2 Genotype and allele distribution of SNPs in DNA repair pathway genes

The genotype and allele frequencies of ten polymorphisms in the DNA repair pathways; *XRCC1* rs25487, *XPD* rs13181, *APE1* rs1130409, *XRCC2* rs3218536, *XRCC3* rs861539, *hOGG1* rs1052133, *XPG* rs17655, *hMSH2* rs4987188, *RAD51* rs2619679, and *RAD51* rs5030789 were determined in BC patients and controls (Table 4). Genotype frequency distributions of *XRCC1* rs25487, *XRCC2* rs3218536, *XRCC3* rs861539, *XPG* rs17655, and *hMSH2* rs4987188 SNPs were found to be statistically different between BC patients and controls. Whereas, no difference was observed in *XPD* rs13181, *APE1* rs1130409, *hOGG1* rs1052133, *RAD51* rs2619679, and *RAD51* rs5030789. A significant difference was determined in allele frequencies of *XRCC1* rs25487, *XRCC3* rs861539, *XPG* rs17655 and *hMSH2* rs4987188 between BC patients and controls (*p* = 0.001, respectively).

**TABLE 4:** Genotype and allele distribution of SNPs in DNA repair pathway genes

SNP	Genotypic frequencies *n* (%)		*p‐*Value		Allelic frequencies		*X* ^*2*^	OR/CI(95%)	*p*‐Value
Genotype	BC patients (*n* = 263)	Control (*n* = 250)		Allele	BC patients (*n* = 263)	Control (*n* = 250)			
*XRCC1*‐Arg399Gln (rs25487)									
AA	175 (66.5)	205 (82)							
AG	75 (28.5)	42 (16.8)	**0.001**	A/G	0.81/0.19	0.90/0.10	17.93	2.18/1.51‐3.16	**0.001**
GG	13 (5)	3 (1.2)							
*XPD*‐Lys751Gln (rs13181)									
GG	35 (13.3)	41 (16.4)							
GT	105 (39.9)	99 (39.6)	0.593	G/T	0.33/0.67	0.35/0.65	0.52	1.10/0.85‐1.42	0.468
TT	123 (46.8)	110 (44)							
*APE1*‐Asp148Glu (rs1130409)									
GG	44 (16.7)	49 (19.6)							
GT	110 (41.8)	106 (42.4)	0.614	G/T	0.38/0.62	0.40/0.60	0.77	1.11/0.87‐1.44	0.397
TT	109 (41.4)	95 (38.0)							
*XRCC2*‐Arg188His (rs3218536)									
AA	234 (89)	209 (83.5)							
AG	29 (11)	41 (16.5)	**0.040**	A/G	0.94/0.16	0.00	1.51	0.73/0.45‐1.20	0.219
GG	0 (0)	0 (0)							
*XRCC3*‐Thr241Met (rs861539)									
CC	149 (56.7)	202 (80.8)							
CT	92 (35)	43 (17.2)	**0.001**	C/T	0.74/0.26	0.89/0.11	38.97	2.91/2.06‐4.11	<**0.001**
TT	22 (8.4)	5 (2)							
*hOGG1*‐Ser326Cys (rs1052133)									
CC	202 (76.8)	201 (80.4)							
CG	53 (20.2)	43 (17.2)	0.606	C/G	0.87/0.13	0.89/0.11	1.08	1.22/0.83‐1.78	0.298
GG	8 (3)	6 (2.4)							
*XPG*‐Asp1104His (rs17655)									
GG	51 (19.4)	104 (41.6)							
GC	121 (46)	107 (42.8)	**0.001**	G/C	0.42/0.58	0.63/0.37	43.63	2.31/1.80 ‐2.97	<**0.001**
CC	91 (34.6)	39 (15.6)							
*hMSH2‐*Gly322Asp (rs4987188)									
GG	207 (78.7)	228 (91.2)							
GA	49 (18.6)	20 (8.0)	**0.001**	G/A	0.88/0.12	0.95/0.5	16.70	2.67/1.64‐4.35	**0.001**
AA	7 (2.7)	2 (0.8)							
*RAD51*‐4719A/T (rs2619679)									
AA	112 (42.6)	121 (48.4)							
AT	124 (47.1)	110 (44)	0.325	A/T	0.66/0.34	0.70/0.30	2.13	1.21/0.93‐1.58	0.144
TT	27 (10.3)	19 (7.6)							
*RAD51*‐4601A/G (rs5030789)									
GG	177 (67.3)	178 (71.2)							
GA	80 (30.4)	67 (26.8)	0.633	G/A	0.83/0.17	0.85/0.15	0.81	1.16/0.83–1.62	0.366
AA	6 (2.3)	5 (2)							

*Note*: The genotype distribution of polymorphisms between the groups was compared using *x* ^*2*^ test. The allelic frequency of polymorphisms between the groups was compared using HWE test. The *p*‐value ≤0.05 considered as statisticlly significant (in bold).

Abbreviations: BC, Breast Cancer; CI, Confidence interval; OR, Odds ratio.

Table 5 shows the results of association analysis between the studied SNPs and risk of BC. For each SNP, the genotypic and allelic association were tested considering multiple inheritance models (dominant, recessive, co‐dominant and additive) (Table 5). Arg (G) allele for *XRCC1* rs25487 (co‐dominant: genotype Gln/Gln (AA) vs. Arg/Arg (GG): OR 4.95, *p* = 0.006, Arg/Arg (GG) vs. Gln/Gln (AA) OR 0.20, *p* = 0.006, dominant OR 2.23, *p* = 0.001, recessive OR 0.23, *p* = 0.016 and additive: OR 1.46 *p* = 0.022) Met (T) allele for *XRCC3* rs861539 (co‐dominant: genotype Thr/Thr (CC) vs. Met/Met (TT): OR 5.90, *p* = 0.001, Met/Met (TT) vs. Thr/Thr (CC) OR 0.16, *p* = 0.001, dominant: OR 3.18, *p* < 0.001, recessive: OR 0.22, *p* = 0.001 and additive: OR 1.48 *p* = 0.001) and His (C) allele for *XPG* rs17655 (co‐dominant: genotype Asp/Asp (GG) vs. His/His (CC): OR 4.75 *p* < 0.001, His/His (CC) vs. Asp/Asp (GG) OR 0.21 *p* < 0.001, dominant: OR 2.96, *p* < 0.001, recessive: OR 0.34, *p* < 0.001 and additive: OR 0.85 *p* = 0.001) showed an association with the increased risk of BC in all tested genetic inheritance models (Table 5). But, Asp (A) allele for *hMSH2* rs4987188 showed an association with BC risk only dominant model (OR 2.77, *p* = 0.001).

**TABLE 5:** Analysis of SNPs based on the four genetic inheritance models

SNP	Model of inheritance	OR (95% CI)	*p*‐Value	AIC^a^
*XRCC1*‐Arg399Gln (rs25487)	**Co‐dominant**			
	AA vs GG	2.23 (1.47–3.37)	**0.006**	—
	GG vs AA	0.23 (0.06–0.84)	**0.006**	—
	**Dominant** AA vs AG + GG	0.67 (0.31–1.30)	**0.001**	15.623
	**Recessive** GG vs AA+AG	1.46 (0.21–2.72)	**0.022**	13.448
	**Additive** AA vs GA vs GG	1.46 (0.21–2.72)	**0.022**	20.309
*XPD*‐Lys751Gln (rs13181)	**Co‐dominant**			
	GG vs TT	1.21 (0.71–2.05)	0.469	—
	TT vs GG	0.82 (0.48–1.39)	0.469	—
	**Dominant** GG vs GT + TT	0.87 (0.10–0.27)	0.364	15.313
	**Recessive** TT vs GG + GT	0.12 (0.03–0.38)	0.358	15.989
	**Additive** GG vs GT vs TT	0.21 (0.14–0.37)	0.395	22.456
*APE1*‐Asp148Glu (rs1130409)	**Co‐dominant**			
	GG vs TT	1.22 (0.74–2.01)	0.419	—
	TT vs GG	0.81 (0.49–1.33)	0.419	—
	**Dominant** GG vs GT + TT	0.08 (0.01–0.27)	0.380	15.478
	**Recessive** TT vs GG + GT	0.13 (0.03–0.41)	0.327	15.954
	**Additive** GG vs GT vs TT	0.11 (0.01–0.42)	0.357	22.583
*XRCC2*‐Arg188His (rs3218536)	**Co‐dominant**			
	AA vs GG	N/A^b^	0.978	—
	GG vs AA	N/A^b^	0.987	—
	**Dominant** AA vs AG + GG	0.34 (0.24–0.82)	0.154	15.221
	**Recessive** GG vs AA+AG	0.05 (0.01–0.22)	0.566	8.692
	**Additive** AA vs GA vs GG	0.36 (0.21–0.84)	0.156	15.244
*XRCC3*‐Thr241Met (rs861539)	**Co‐dominant**			
	CC vs TT	5.90 (2.18–15.95)	**0.001**	—
	TT vs CC	0.16 (0.06–0.45)	**0.001**	—
	**Dominant** CC vs CT + TT	0.88 (0.54–1.22)	**0.001**	15.640
	**Recessive** TT vs CC + CT	1.09 (0.86–2.11)	**0.033**	13.849
	**Additive** CC vs CT vs TT	1.48 (0.51–2.45)	**0.001**	20.778
*hOGG1*‐Ser326Cys (rs1052133)	**Co‐dominant**			
	CC vs GG	1.32 (0.45–3.89)	0.605	—
	GG vs CC	1.22 (0.74–2.01)	0.605	—
	**Dominant** CC vs CG + GG	0.22 (0.15–0.60)	0.254	15.596
	**Recessive** GG vs CC + CG	0.29 (0.07–1.34)	0.594	13.771
	**Additive** CC vs CG vs GG	0.31 (0.08–1.36)	0.596	20.569
*XPG*‐Asp1104His (rs17655)	**Co‐dominant**			
	GG vs CC	4.75 (2.87–7.86)	**0.006**	—
	CC vs GG	0.21 (0.12–0.34)	**0.006**	—
CC vs GG + GC	**Dominant** GG vs GC + CC	0.37 (0.16–0.58)	**0.001**	15.673
GG vs cc	**Recessive** CC vs GG + GC	0.84 (0.45–1.22)	**0.002**	15.524
CC vs GG	**Additive** GG vs GC vs CC	0.85 (0.47–1.73)	**0.001**	22.403
hMSH2‐Gly322Asp (rs4987188)	**Co‐dominant**			
	GG vs AA	3.82 (0.78–18.60)	0.074	—
	AA vs GG	0.26 (0.05–1.27)	0.076	—
	**Dominant** GG vs GA + AA	0.93 (0.44–1.42)	**0.001**	15.132
	**Recessive** AA vs GG + GA	1.25 (0.31–2.82)	0.118	13.043
	**Additive** GG vs GA vs AA	1.26 (0.32–2.84)	0.119	19.394
*RAD51*‐4719A/T (rs2619679)	**Co‐dominant**			
	AA vs TT	1.53 (0.80–2.91)	0.187	—
	TT vs AA	0.65 (0.34–1.23)	0.187	—
	**Dominant** AA vs AT+TT	0.15 (0.07–0.39)	0.189	15.985
	**Recessive** TT vs AA+AT	0.37 (0.23–0.96)	0.23	14.817
	**Additive** AA vs AT vs TT	0.35 (0.23–0.93)	0.241	22.07
*RAD51*‐4601A/G (rs5030789)	**Co‐dominant**			
	GG vs AA	1.20 (0.36–4.02)	0.759	—
	AA vs GG	0.82 (0.24–2.76)	0.759	—
	**Dominant** GG vs GA + AA	0.18 (0.13–0.50)	0.266	15.835
	**Recessive** AA vs GG + GA	0.18 (0.04–1.37)	0.763	13.558
	**Additive** GG vs GA vs AA	0.19 (0.04–1.36)	0.764	20.65

*Note*: The *p*‐value ≤0.05 was considered as statistically significant. The *p*‐values in bold remained significant after Bonferroni correction.

Abbreviations: AIC, Akaike's information criterion; CI, Confidence interval; N/A, not available; OR, Odds ratio.

^a^ The AIC: the preferred inheritance model is the one with the minimum AIC value.

^b^ Unable to calculate since the CC genotype was absent in the BC patients and control group.

### 3.3 Haplotype analysis of the DNA repair genes polymorphisms

Association analysis between the risk of BC and haplotypes of ten polymorphisms was determined by Haploview 4.2, the results are summarized in Table 6. The haplotypes were generated using ten DNA repair gene polymorphisms (*hOGG1* rs1052133/*APE1* rs1130409/*RAD51* rs2619679/*RAD51* rs5030789/*XRCC1* rs25487*/XPD* rs13181/*hMSH2* rs4987188/*XPG* rs17655/*XRCC3* rs861539/*XRCC2* rs3218536). We observed that, the frequency of CTTGAGGCCA (*p* = 0.0249), CTTGATGCTA (*p* = 0.0022), and CTAGATGCTA (*p* = 0.0331) haplotypes were significantly higher in BC patients than controls. Also, these haplotypes could be linked with a significant increased (high‐risk haplotypes) BC risk. Whereas, the frequency of CGAGAGGGCA (*p* = 0.0031), CTAGATGGCA (*p* = 0.0204), and CTTGAGGGCA (*p* = 0.0332) haplotypes were found to be significantly decreased in BC patients than controls. These haplotypes might be well associated with a significant reduced (low‐risk haplotypes) BC risk in Tanzania.

**TABLE 6:** Frequencies of haplotypes of DNA repair genes in the study group

Haplotype Associations	Frequency	BC patients	Controls	*Χ* ^*2*^	*p*‐Value
*hOGG1/APE1/RAD51‐4719A/T/RAD51‐4601A/G/XRCC1/XPD hMSH2/XPG/ XRCC3/XRCC2*					
CTAGATGCCA	0.100	0.107	0.098	0.22	0.6394
CGAGATGGCA	0.082	0.067	0.102	3.979	**0.0461**
CTAGAGGCCA	0.058	0.060	0.059	0.01	0.9186
CGAGAGGGCA	0.045	0.027	0.067	8.763	**0.0031**
CGTGATGGCA	0.038	0.032	0.045	1.156	0.2823
CTAGATGGCA	0.037	0.024	0.052	5.377	**0.0204**
CTTGATGGCA	0.026	0.017	0.037	3.869	**0.0492**
CTTGATGCCA	0.023	0.017	0.029	1.575	0.2095
CTTGAGGCCA	0.020	0.031	0.010	5.029	**0.0249**
CTTGATGCTA	0.016	0.028	0.004	9.405	**0.0022**
CTAGATGCTA	0.014	0.023	0.006	4.543	**0.0331**
CGAGGTGGCA	0.013	0.016	0.011	0.362	0.5471
CTTGAGGGCA	0.013	0.006	0.021	4.537	**0.0332**
CGAGATGCCA	0.013	0.015	0.011	0.408	0.5232
CTTAATGCCA	0.012	0.010	0.015	0.596	0.4402
CTTGGTGCCA	0.012	0.014	0.010	0.258	0.6118
CGTGATGGTA	0.011	0.017	0.006	2.503	0.1136
GGAGATGGTA	0.011	0.009	0.013	0.397	0.5287
GTAGATGCCA	0.010	0.011	0.010	0.017	0.897
CTAGGGGCCA	0.010	0.013	0.008	0.759	0.3837

*Note*: The *p*‐value ≤0.05 was considered as statistically significant (in bold).

### 3.4 Genotypes stratified by menopausal status and age at the time of BC diagnosis

The association between the SNPs and BC risk stratified by menopausal status and age at BC diagnosis of the patients are summarized in Table 7. The results revealed that *XRCC1*‐Gln/Arg (AG) was the most frequent genotype with 3.1‐fold increased risk of developing BC in pre‐menopausal patients than their post‐menopausal counterparts (OR = 3.23, CI 95% = 2.08–5.02, *p* = 0.001).

**TABLE 7:** Genotypes stratified by menopausal status and age at the time of BC diagnosis

SNP	Pre‐menopausal BC patients *n* (%)	Post‐menopausal BC patients *n* (%)	*p*‐value	Age at the time of BC diagnosis >40 *n* (%)	Age at the time of BC diagnosis <40	*p*‐value
					*n* (%)	
*XRCC1*‐Arg399Gln (rs25487)						
AA	55 (46.2)	120 (83.3)		81 (64.3)	94 (68.6)	
AG	57 (47.9)	18 (12.5)	**0.001**	39 (31)	36 (26.3)	0.578
GG	7 (5.9)	6 (4.2)		6 (4.7)	7 (5.1)	
*XPD‐*Lys751Gln (rs13181)						
GG	14 (11.8)	21 (14.6)		12 (9.5)	23 (16.8)	
GT	49 (41.2)	56 (38.9)	0.699	56 (44.4)	49 (35.8)	0.499
TT	56 (47)	67 (46.5)		58 (46.1)	65 (47.4)	
*APE1*‐Asp148Glu (rs1130409)						
GG	19 (16)	25 (17.4)		17 (13.5)	27 (19.7)	
GT	48 (40.3)	62 (43.1)	0.539	49 (38.9)	61 (44.5)	0.120
TT	52 (43.7)	57 (39.6)		60 (47.6)	49 (35.8)	
*XRCC2*‐Arg188His (rs3218536)						
AA	107 (89.9)	127 (88.2)		111 (88.1)	123 (89.8)	
AG	12 (10.1)	17 (11.8)	0.405	15 (11.9)	14 (10.2)	0.405
GG	0 (0)	0 (0)		0 (0)	0 (0)	
*XRCC3*‐Thr241Met (rs861539)						
CC	71 (59.7)	78 (54.2)		74 (58.7)	75 (54.7)	
CT	38 (31.9)	54 (37.5)	0.498	43 (34.1)	49 (35.8)	0.428
TT	10 (8.4)	12 (8.3)		9 (7.1)	13 (9.5)	
*hOGG1*‐Ser326Cys (rs1052133)						
CC	95 (79.8)	107 (74.3)		100 (79.4)	102 (74.5)	
CG	22 (18.5)	31 (21.5)	0.201	23 (18.3)	30 (21.9)	0.322
GG	2 (1.7)	6 (4.2)		3 (2.3)	5 (3.6)	
*XPG*‐Asp1104His (rs17655)						
GG	24 (20.2)	27 (18.8)		18 (14.3)	33 (24.1)	
GC	52 (43.7)	69 (47.9)	0.877	58 (46)	63 (46)	**0.028**
cc	43 (36.1)	48 (33.3)		50 (39.7)	41 (29.9)	
*hMSH2*‐Gly322Asp (rs4987188)						
GG	96 (80.7)	111 (77.1)		96 (76.2)	111 (81)	
GA	21 (17.6)	28 (19.4)	0.372	28 (22.2)	21 (15.3)	0.644
AA	2 (1.7)	5 (3.5)		2 (1.6)	5 (3.7)	
*RAD51*‐4719A/T (rs2619679)						
AA	48 (40.3)	64 (44.4)		57 (45.2)	55 (49.1)	
AT	57 (47.9)	67 (46.5)	0.397	55 (43.7)	69 (50.4)	0.667
TT	14 (11.8)	13 (9)		14 (11.1)	13 (9.5)	
*RAD51*‐4601A/G (rs5030789)						
GG	72 (60.5)	105 (72.9)		83 (65.9)	94 (68.6)	
GA	43 (36.1)	37 (25.7)	0.085	41 (32.5)	39 (28.5)	0.828
AA	4 (3.4)	2 (1.4)		2 (1.6)	4 (2.9)	

*Note*: The genotype distribution of polymorphisms between the groups was compared using x^2^ test. The *p*‐value ≤0.05 was considered as statistically significant (in bold).

Abbreviation: BC, Breast Cancer.

In order to examine the association between the SNPs and the age at BC diagnosis, we subgrouped the patients according to the median age at diagnosis (median age at diagnosis = 40 years); age at the time of BC diagnosis ≥40 years and age at the time of BC diagnosis <40 years. The analyses showed that *XPG* rs17655 SNP was significantly associated with BC patients who had been diagnosed at younger ages (<40 years). We observed that the His/His (CC) genotype had a 1.2‐fold increased risk of BC in patients diagnosed at younger ages (<40 years) than those diagnosed with the disease at older ages (≥40 years) (OR = 0.69, CI 95% = 0.50–0.96, *p* = 0.028) (Table 7).

### 3.5 Genotypes and histopathological characteristics of breast tumors

Histopathologic characteristics of the breast tumors are summarized in Table 2. PR positivity and Luminal‐A subtype were significantly associated with *XPG* rs17655 (*p* = 0.042 and *p* = 0.021, respectively) (Table 8). Also, Asp/His+ His/His (GC + CC) genotypes were found to increase the risk of developing BC around 1.3‐fold in PR+ patients compared to PR– counterparts (OR = 0.45, CI 95% = 0.24–0.85, *p* = 0.014), whereas Asp/His+His/His (GC + CC) genotypes decreased risk of developing BC 1.1‐fold in Luminal‐A subtype patients than non‐Luminal‐A counterparts (OR = 0.46, CI 95% = 0.23–0.87, *p* = 0.020). Moreover, HER2 enriched subtype was significantly associated with *hMSH2* rs4987188 (*p* = 0.028) (Table 8), and Gly/Asp (GA) genotype decreased risk of developing BC almost 6‐fold in HER2 enriched subtype patients compared to non‐HER2 enriched subtype counterparts (OR = 0.47, CI 95% = 0.26–0.94, *p* = 0.033). No relationship was detected between other SNP and histopathological characteristics of breast tumors.

**TABLE 8:** Association between genotypes and histopathological characteristics of breast tumors

SNP	PR+ *n* (%)	PR‐ *n* (%)	*p*‐Value	Luminal‐A *n* (%)	Non‐Luminal‐A *n* (%)	*p*‐Value	HER‐2 *n* (%)	Non‐HER2 *n* (%)	*p*‐Value
*XRCC1*‐Arg399Gln (rs25487)									
AA	90 (64.7)	85 (68.5)		79 (68.1)	96 (65.3)		18 (58)	157 (67.7)	
AG	43 (30.9)	32 (25.8)	0.730	32 (27.6)	43 (29.3)	0.586	10 (32.3)	65 (28)	0.177
GG	6 (4.4)	7 (5.7)		5 (4.3)	8 (5.4)		3 (9.7)	10 (4.3)	
*XPD*‐Lys751Gln (rs13181)									
GG	14 (10.1)	21 (16.9)		15 (12.9)	20 (13.6)		2 (6.5)	33 (14.2)	
GT	56 (40.3)	49 (39.5)	0.134	48 (41.4)	57 (38.8)	0.885	14 (45.1)	91 (39.2)	0.473
TT	69 (49.6)	54 (43.6)		53 (45.7)	70 (47.6)		15 (48.4)	108 (46.6)	
*APE1*‐Asp148Glu (rs1130409)									
GG	15 (10.8)	29 (23.4)		14 (12)	30 (20.4)		8 (25.8)	36 (15.5)	
GT	65 (46.8)	45 (36.3)	0.099	51 (44)	59 (40.1)	0.152	15 (48.4)	95 (40.9)	0.121
TT	59 (42.4)	50 (40.3)		51 (44)	58 (39.5)		8 (25.8)	101 (43.6)	
*XRCC2*‐Arg188His (rs3218536)									
AA	121 (87.1)	113 (91.1)		101 (87.1)	133(90.5)		28 (90.3)	206 (88.8)	
AG	18 (12.9)	11 (8.9)	0.329	15 (12.9)	14 (9.5)	0.248	3 (9.7)	26 (11.2)	0.545
GG	0 (0)	0 (0)		0 (0)	0 (0)		0 (0)	0 (0)	
*XRCC3*‐Thr241Met (rs861539)									
CC	77 (55.4)	72 (58.1)		67 (57.8)	82 (55.8)		20 (64.5)	129 (55.6)	
CT	47 (33.8)	45 (36.3)	0.328	37 (31.9)	55 (37.4)	0.845	9 (29)	83 (35.8)	0.370
TT	15 (10.8)	7 (5.6)		12 (10.3)	10 (6.8)		2 (6.5)	20 (8.6)	
*hOGG1*‐Ser326Cys (rs1052133)									
CC	106 (76.3)	96 (77.4)		88 (75.9)	114 (77.6)		26 (83.9)	176 (75.9)	
CG	29 (20.9)	24 (19.4)	0.896	25 (21.6)	28 (19)	0.889	4 (12.9)	49 (21.1)	0.420
GG	4 (2.8)	4 (3.2)		3 (2.5)	5 (3.4)		1 (3.2)	7 (3)	
*XPG*‐Asp1104His (rs17655)									
GG	19 (13.7)	32 (20.6)		15 (12.9)	36 (24.5)		9 (29)	42 (18.1)	
GC	68 (48.9)	53 (47.9)	**0.042**	55 (47.4)	66 (44.9)	**0.021**	15 (48.4)	106 (45.7)	0.075
CC	52 (37.4)	39 (31.5)		46 (39.7)	45 (30.6)		7 (22.6)	84 (36.2)	
*hMSH2*‐Gly322Asp (rs4987188)									
GG	111 (79.9)	96 (77.4)		96 (82.8)	111 (75.5)		21 (67.7)	186 (80.2)	
GA	26 (18.7)	23(18.5)	0.402	18(15.5)	31(21.2)	0.139	7(22.6)	42(18.1)	**0.028**
AA	2 (1.4)	5 (4.1)		2 (1.7)	5 (3.4)		3 (9.7)	4 (1.7)	
*RAD51*‐4719A/T (rs2619679)									
AA	63 (45.3)	49 (39.5)		52 (44.8)	60 (40.8)		12 (38.7)	100 (43.1)	
AT	66 (47.5)	58 (46.8)	0.126	55 (47.4)	69 (46.9)	0.294	13 (41.9)	111 (47.8)	0.239
TT	10 (7.2)	17 (13.7)		9 (7.8)	18 (12.3)		6 (19.4)	21 (9.1)	
*RAD51*‐4601A/G (rs5030789)									
GG	93 (66.9)	84 (67.7)		78 (67.2)	99 (67.3)		22 (71)	155 (66.8)	
GA	42 (30.2)	38 (30.6)	0.745	35 (30.2)	45 (30.6)	0.920	9 (29)	71 (30.6)	0.501
AA	4 (2.9)	2 (1.7)		3 (2.6)	3 (2.1)		0 (0)	6 (2.6)	

*Note*: The genotype distribution of polymorphisms between the groups was compared using *x* ^*2*^ test. The *p*‐value≤0.05 considered as statistically significant (in bold).

Abbreviations: HER‐2, Human epidermal growth factor receptor‐2; PR, Progesterone receptor.

### 3.6 Gene–gene and gene–family history of cancer in first‐degree relatives interactions on BC risk

We performed a data‐mining analytical approach MDR to explore the potential gene–gene interaction and gene‐family history of cancer in first‐degree relatives interaction. Each overall best model of all quantities was weighed by testing accuracy and cross‐validation consistency. Three models were built and they included the BC associated SNPs of our study and family history of cancer in their first‐degree relatives, and these models inferred by the method are shown in Table 9.

**TABLE 9:** Multifactor dimensionality reduction (MDR) analysis for the breast cancer risk predication

Best Model	CVC	Testing accuracy	Permutation test *p* value^a^
One‐Factor *XPG‐*Asp1104His (rs17655)	10/10	0.6247	**<0.001**
Two‐Factor *XPG*‐Asp1104His (rs17655) and family history of cancer in first‐degree relatives	8/10	0.6518	**<0.001**
Three‐Factor *XRCC3*‐Thr241Met (rs861539), *XPG*‐Asp1104His (rs17655) and family history of cancer in first‐degree relatives	10/10	0.7108	**<0.001**

*Note*: The *p*‐value≤0.05 considered as statistically significant (in bold).

Abbreviation: OR, Odds ratio.

^a^ 1000‐fold permutation test.

**FIGURE 1:**
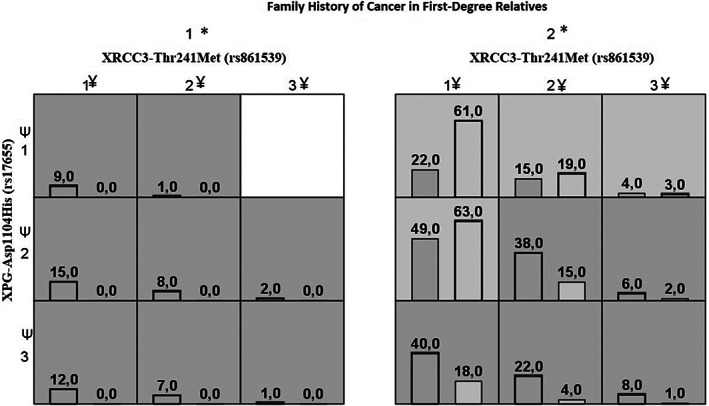
XRCC3‐Thr241Met (rs861539), XPG‐Asp1104His (rs17655) and family history of cancer in first‐degree relatives combined are associated with high and low risks of breast cancer multifactor dimensionality reduction (MDR) analysis with the highest testing accuracy. For each multifactor cell, the number of breast cancer cases is displayed in the left bar and the number of controls is displayed in the right bar. Cells of dark gray indicated high risk combinations and cells of light gray indicated low risk combinations. Family history of cancer in first‐degree relatives: 1* positive, 2* negative; XRCC3‐Thr241Met (rs861539): 1^¥^ Thr/Thr (CC) genotype, 2^¥^ Thr/Met (CT) genotype, 3^¥^ Met/Met (TT) genotype; XPG‐Asp1104His (rs17655): 1^Ψ^ Asp/Asp (GG) genotype, 2^Ψ^ Asp/His (GC) genotype, 3^Ψ^ His/His (CC) genotype

It is well known that family history of cancer in first‐degree relatives is the major risk factor of BC. MDR results showed that family history of cancer in first‐degree relatives was emerged in the best model. The best one‐factor model for BC risk predication included *XPG* rs17655, with the highest cross‐validation consistency (CVC) of 10/10 and testing accuracy of 63.47%. In two‐factors model, *XPG* rs17655 and family history of cancer in first‐degree relatives, was the best two‐factor predictors of BC risk, with the highest CVC of 8/10 and testing accuracy of 65.18%, which was higher than that of the one‐factor model. Thus, showed slightly improved capability of prediction than *XPG* rs17655 but a decrease in CVC. The three‐factors model consisted of *XRCC3* rs861539, *XPG* rs17655 and family history of cancer in first‐degree relatives and it was the strongest and best model with a CVC of 10/10 and the highest testing accuracy of 71.08%. Compared with the best of one‐or‐three factor models, the best of the three‐factor model consisting of *XRCC3* rs861539, *XPG* rs17655 and family history of cancer in first‐degree relatives had improved testing accuracy and CVC, and it was thought to be the fitted model (Figure 1).

**FIGURE 2:**
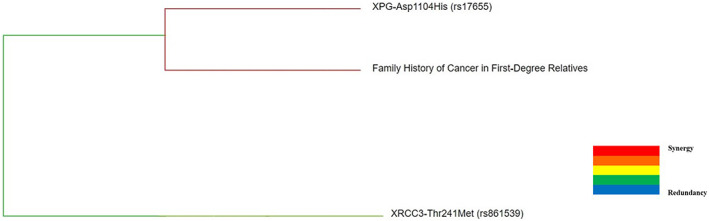
Interaction dendrogram gained from the multifactor dimensionality reduction (MDR) for gene–gene and gene‐family history of cancer in first‐degree relatives interactions on breast cancer risk. XPG‐Asp1104His (rs17655) and family history of cancer in first‐degree relatives had the strongest synergistic interaction

The interaction dendrogram was created with MDR to demonstrate the visualized interaction of these SNPs and family history of cancer in first‐degree relatives (Figure 2). The dendrogram placed the attributes that have strong interaction close together at the leaves of the tree. The colors of the branch indicated the degree of interaction from strong to weak (red, orange, gold, green and blue) as follows; red represented the highest degree of interaction or synergy, and green represented low interaction. The interaction dendrogram showed that there were synergistic interactions between *XPG* rs17655 and family history of cancer in first‐degree relatives. Moreover, interaction dendrogram placed *XPG* rs17655 and family history of cancer in first‐degree relatives on the same branch, but *XRCC3* rs861539 on another branch.

## 4 DISCUSSION

Human cells employ DNA repair mechanism(s) to correct the damages that would otherwise accumulate and eventually cause genetic instability and cancer. The genetic variations in the DNA repair genes undergird the variation in the DNA repair capacity among the population.^14^, ^15^ The variation in DNA repair capacity may be disadvantageous to individuals with reduced capacity, thereby increasing their susceptibility to various cancers.^16^ The knowledge pertaining the interaction of these genetic variants in the DNA repair pathways with reproductive factors, which are well‐known risk factors for the development of BC is very limited. Thus, in this study, we focused on the association of notable polymorphisms residing in the DNA repair genes with BC in the Tanzanian population and possible relationship of the polymorphisms and reproductive risk factors of BC.

The DNA repair genes harboring the polymorphisms examined in this study are involved in the following repair pathways: BER (*XRCC1* rs25487, *APE1* rs1130409, *hOGG1* rs1052133), NER (*XPG* rs17655, *XPD* rs13181), DSBR (*XRCC2* rs3218536, *XRCC3* rs861539, *RAD51* rs2619679, *RAD51* rs5030789), and MMR (*hMSH2* rs4987188). Among those polymorphisms, the followings were found to be significantly associated with BC in Tanzanian population: *XRCC1* rs25487, *XPG* rs17655, *XRCC2* rs3218536, *XRCC3* rs861539, and *MSH2* rs4987188.

The *XRCC1* gene codes an important protein that coordinates the assembly and interactions of other proteins required for BER.^17^ The common polymorphism of *XRCC1* is rs 25,487 which is theorized to affect the interaction of its protein with Poly (ADP‐ribose) polymerase (PARP) and glycolases components of the BER machinery.^17^ An in‐vitro study utilizing cultured lymphoblasts comparing DNA repair abilities between *XRCC1* 399Gln and 399Arg allelic variants showed that 399Gln variant has a reduced DNA repair ability.^18^ The result of our study showed that the *XRCC1* rs25487 (Arg/Gln and Gln/Gln genotypes and 399Gln allele) polymporphism was significantly associated with BC risk in the Tanzanian women. This was in agreement with the findings from other ethnic populations.^19^, ^20^, ^21^, ^22^ However, contrast to our findings, a study in Russian women indicated that 399Arg codon to be a potential risk factor for BC.^23^ Some other studies, however, have found no association between *XRCC1* rs25487 polymorphism and BC susceptibility.^24^, ^25^ We also demostrated that three genetic inheritance models of *XRCC1* rs25487 polymorhism were associated with BC risk in our study population. Nevertheless, these results are supported by a meta analysis study that suggested the dominant model (Arg/Gln + Arg/Arg vs Gln/Gln) and co‐dominant model (Gln/Gln vs. Arg/Arg, and Arg/Arg vs. Gln/Gln) were associated with BC in African population. Though, the recessive model (Gln/Gln vs Arg/Gln + Arg/Arg) and co‐dominant model were reported to be associated with BC in Asian population,^26^ besides in a single study, the recessive model was reported to increase BC risk in Indian and African population.^27^

The *XPG* gene is a major gene that encodes a key protein that interacts with other proteins to bring about the NER pathway. The polymorphism in the codon 1104 that substitutes His for Asp in the protein is among the genetic variations in the gene that affects its NER capacity.^28^ In this study, we found that His/His genotype and 1104His allele were significantly associated with increased risk of BC among Tanzanian women. Our results confirmed the significant associations of *XPG* rs17655 with the risk of BC reported in Taiwanese and Finnish populations.^29^, ^30^ Moreover, three genetic inheritance models of *XPG* rs17655 polymorphism were found to be associated with BC risk in our study. However, a lack of association between this polymorphism and cancer has been reported from other studies.^31^, ^32^, ^33^

XRCC2 and XRCC3 work with RAD51 and other RAD51 paralog proteins to mediate homologous recombination that repairs DNA DSB.^34^ Unrepaired DSB can result in the formation of micronuclei, and these have been used as biomarkers for chromosomal stability. A link between *XRCC3* rs861539 polymorphism and micronucleus formation likelihood following exposure to clastogenic agents has been investigated, with the 241Met variant being associated with higher levels of micronuclei among formaldehyde‐exposed workers in *Laidera* et al. study.^35^ As high levels of micronuclei reflect weak DSB repair, this study's findings are in agreement with our results, associating Met allele with BC. In accordance with our results, a study from China suggested that Met/Met genotype and 241Met allele were associated with BC risk.^36^ However, these results are conflicting with the findings from *Mateuca* et al.^37^ that reported high frequencies of micronuclei in individuals carrying Thr/Thr or Thr/Met genotypes, attributing weak DSB capacity to Thr allele. Nonetheless, there exists some studies that could not find an association between *XRCC3* rs861539 and BC risk.^23^, ^38^ Furthermore, we found that three genetic inheritance models of *XRCC3* rs861539 were associated with BC in our study group. The *XRCC2* rs3218536 polymorphism was also found to be associated with BC in our study. The Arg/His genotype was more frequent in control group than BC patients, therefore we can suggest that *XRCC2* rs3218536 polymorphism (Arg/His genotype and 188His allele) could be a protective polymorphism for BC in Tanzanian women. The similar results were observed in a case–control study in Caucasian and Portuguese populations. Authors speculated that *XRCC2* rs3218536 polymorphism decreased risk of BC in women at post‐menopausal status and never breastfed.^39^

The hMSH2 protein play an integral part in repairing mismatched nucleotides following DNA replication.^40^ We examined the relationship between *hMSH2* rs4987188 polymorphism and BC. We observed that Gly/Asp and Asp/Asp genotypes and 322Asp allele were more frequent in BC cases than the controls. Also, the dominant model of *hMSH2* rs4987188 polymorphism (Gly/Gly vs Gly/Asp+Asp/Asp) was strongly associated with BC in Tanzanian women. Our results show the possible polymorphic effect of this polymorphism on cancer susceptibility, with variant 322Asp linking to BC in a dominant inheritence model. Our findings agree with *Smolarz* et al. that reported a significant association between 322Asp variant and BC susceptibility.^22^ However, the study by *Poplawski* et al. reported conflicting results, attributing 322Gly variant to BC susceptibility^41^ while the meta‐analysis by *Zhang* et al. finds no observable association between *hMSH2* rs4987188 polymorphism and BC.^42^

Moreover, we analyzed the possible interaction of BC reproductive risk factors and studied polymorphisms. Our results show that *XPG* rs17655 is more prevalent in younger BC patients and His/His genotype had a 1.2‐fold increased risk of BC in younger (<40 years) patients. The *XRCC1* rs25487 polymorhism was found to be associated with the menopausal status. The Arg/Gln genotype of the *XPG* rs17655 polymorphism had a 3.1‐fold increased risk of BC in pre‐menopausal patients than their post‐menopausal counterparts. Recent studies in different populations also reported the association between *XRCC1* rs25487 polymorphism and menopausal status. In agreement with our results, a study in Indian populations showed that *XRCC1* rs25487 polymorphism was 6‐fold higher in pre‐menopausal BC patients.^20^ A study in Caucasian Portuguese population and a recently published meta‐analysis suggested that Gln/Gln genotype increased risk of BC in post‐menopause women aged over 55 years.^43^, ^44^ In addition, *XPG* rs17655 and *hMSH2* rs4987188 polymorphisms were found to be associated with histopathological characteristics of breast tumors. Asp/His+His/His genotypes had a 1.3‐fold increased risk of BC in PR+ patients and a 1.1‐fold decreased risk of BC in luminal‐A subtype patients when compared to their counterparts. The *hMSH2* rs4987188 polymorphism was significantly associated with HER2‐enriched subtype. The Gly/Asp genotype had almost a 6‐fold decreased risk of BC in HER2‐enriched subtype patients when compared to their counterparts.

The associations between BC risk and haplotypes of studied polymorphisms of DNA repair genes (*hOGG1* rs1052133/*APE1* rs1130409/ *RAD51* rs2619679/*RAD51* rs5030789/*XRCC1* rs25487/*XPD* rs13181/*hMSH2* rs4987188/*XPG* rs17655/*XRCC3* rs861539/*XRCC2* rs3218536) were assessed in this study. Based on the analysis, the frequencies of CTTGAGGCCA, CTTGATGCTA and CTAGATGCTA haplotypes were higher in the controls, whereas the CGAGAGGGCA, CTAGATGGCA and CTTGAGGGCA haplotypes were at lower frequency. Hence, we inferred that the CTTGAGGCCA, CTTGATGCTA and CTAGATGCTA haplotypes may play an important role in decreasing the BC risk, whereas the CTTGAGGCCA, CTTGATGCTA and CTAGATGCTA haplotypes were increasing the risk of BC in Tanzanian women.

We further analyzed the interaction between gene–family history of cancer in the first‐degree relatives by the use of MDR method in BC patients and controls. We identified a statistically significant best model from ten polymorphisms of nine DNA repair genes. Family history of cancer in first‐degree relatives and *XPG* rs17655 built the best model with testing accuracy of 65.18% and CVC of 8/10. The results indicate that the *XPG* rs17655 polymorphisms and their interaction with family history of cancer in the first‐degree relatives might well contribute to BC risk in Tanzanian women. Moreover, the dendrogram (Figure 2) showed that there were synergistic interactions between *XPG* rs17655 and family history of cancer in the first‐degree relatives. Based on the MDR analysis and dendrogram results, the interaction of *XPG* rs17655 and family history of cancer in the first‐degree relatives was found to be significantly synergistic, and this interaction could be responsible for BC risk in Tanzanian population.

Although there exist confling results on DNA repair genes polymorphism and BC, both theoretical and experimental evidences showed the possiblity of these genetic component in pathogenesis of BC and other cancers.^21^, ^22^, ^23^, ^32^ It is posssible that genetic variants uderpinning the development of BC are not the same in all individuals as well as in different populations. And probably a particular population may be characterized by a certain set of genetic variations specific for their population. Nevertheless, this does not underestimate the fact that BC is multifactorial disease caused by genetic, environmental factors and reproductive factors.^2^, ^6^ Thus, the investigations of the mechanism of BC development should be expanded from genetic mechanisms to gene–environment‐reproductive factors interactions.

We designed our study to investigate the potential role of DNA repair gene polymorphisms on the development of BC and possible interaction of these polymorphisms with reproductive factors in Tanzanian BC patients. We found that *XRCC1* rs25487, *XPG* rs17655, *XRCC3* rs861539 and *hMSH2* rs4987188 polymorphisms are potential DNA repair genetic contributors in BC development, whereas *XRCC2* rs3218536 polymorphism could be protective for the BC development among Tanzanian population. Based on the haplotype analysis, the haplotypes CTTGAGGCCA, CTTGATGCTA and CTAGATGCTA can potentially serve as genetic markers for BC susceptibility among women in Tanzania. Interestingly, the *XPG* rs17655 polymorphism could play more active role as it might exert both independent and interactive effects on the development of BC in Tanzanian women.

It is worth noting that our study had a few limitations including participants recruitment criteria. BC patients reported in this study had received breast surgery elsewhere, and were at either chemotherapy, endocrine therapy, radiotherapy or combination treatment at ORCI. Differences of BC treatment modalities between patients could have an impact to our results. Our study missed the representation of women with limited cancer awareness and those who could not reach the facility for various reasons. Also, our study did not take into consideration the survivorship bias between BC subtypes. It is well established that BC patients of triple‐negative subtype have the poorest survival.^13^

In conclusion, our study indicated that the *XRCC1* rs25487, *XPG* rs17655, *XRCC3* rs861539 and *hMSH2* rs4987188 were associated with the BC risk in Tanzanian women. To the best of our knowledge, this is the first study to demonstrate the *XPG* rs17655 polymorphism interaction with not only reproductive factors (such as menopausal status), but also with histopathological characteristics of breast tumors and family history of cancer in the first‐degree relatives. These findings call attention for the investigators not only to focus on the genetic variations in the DNA repair genes, but also the interactions of the genetic variations with reproductive factors. Moreover, understanding of these mechanisms would contribute to the improvement of prognosis and prevention of BC.

## AUTHOR CONTRIBUTIONS

Ismael C. Adolf: Conceptualization, Investigation, Methodology, Writing‐Original draft; Linus P. Rweyemamu: Conceptualization, Investigation, Methodology, Writing‐Original draft, Writing‐review and editing; Gokce Akan: Conceptualization, Investigation, Methodology, Data curation, Formal analysis, Visualization, Writing‐Original draft, Writing‐review and editing; Ted F. Mselle: Methodology, Writing‐review and editing; Nazima Dharsee: Resources, Writing‐review and editing; Lucy A. Namkinga: Supervision, Writing‐review and editing; Sylvester L. Lyantagaye: Supervision, Writing‐review and editing; Fatmahan Atalar: Conceptualization, Methodology, Project administration, Resources, Validation, Supervision, Writing‐review and editing.

## FUNDING INFORMATION

No specific funding to declare.

## CONFLICT OF INTEREST

Authors declare no conflict of interests.

## ETHICAL APPROVAL STATEMENT

This study was approved by the Institutional Review Board of the Ocean Road Cancer Institute (10/Vol/XX/16), and the National Institute for Medical Research (NIMR/HQ/R.8a/Vol.IX/3255). Written informed consent was obtained from all participants.

## Data Availability

The data generated and/or analyzed during the present study are available from the corresponding author on reasonable request.
